# Association of anxiety and depression symptoms with perceived health risk of nicotine vaping products for smoking cessation

**DOI:** 10.3389/fpsyt.2024.1277781

**Published:** 2024-02-29

**Authors:** Joshua Trigg, Ryan Calabro, Patrick Anastassiadis, Jacqueline Bowden, Billie Bonevski

**Affiliations:** ^1^ Flinders Health and Medical Research Institute, College of Medicine and Public Health, Flinders University, Adelaide, SA, Australia; ^2^ Behavioural Research Unit, Cancer Council SA, Adelaide, SA, Australia; ^3^ National Centre for Education and Training on Addiction, Flinders University, Bedford Park, SA, Australia

**Keywords:** vaping, smoking, mental health, prescription access, Australia, E-cigarette (e-cig), anxiety, depression

## Abstract

As tobacco smoking prevalence is unacceptably high for the one in five Australians reporting a mental health condition in the past year, multiple cessation supports are needed to reduce tobacco-related disease. Nicotine vaping product (NVP)-facilitated smoking cessation is one option requiring a medical prescription in Australia. Yet, people easily obtain NVPs via non-prescription channels. As mental health impacts quitting intentions and health system engagement, this study examined how presence of anxiety and depression symptoms may be associated with perceived health risk of using NVPs from prescription or non-prescription sources for smoking cessation. We used cross-sectional South Australian (15 years +) 2022 survey data on vaping, smoking, anxiety, and depression. Robust linear regression was used to examine the association of anxiety and depression symptoms and nicotine addiction concern on perceived health risk of using NVPs from prescription or non-prescription sources. For prescription NVPs, vaping was associated with lower perceived health risk (b=−0.732). Higher perceived addiction risk was associated with higher perceived health risk from prescription NVPs (b=0.784). For non-prescription NVPs, vaping (b=−0.661) or smoking (b=−0.310) was associated with lower perceived health risk, and higher perceived addiction risk (b=0.733) was associated with a higher perceived health risk. Although anxiety and depression were not directly associated with NVP health risk perceptions, vaping while having depression symptoms was associated with higher perceived health risk ratings for prescription (b=0.700) but not non-prescription sources. People with depression who vape may see health risk barriers in NVP prescription access for smoking cessation, a smoking cessation support gap.

## 1 Introduction

Despite successful tobacco control efforts in Australia, smoking rates continue to be unacceptably high at 11% in the general population and up to 84% among some population groups (1). There is high-level evidence of the effectiveness of nicotine vaping products (NVP) to assist people to stop smoking (2). Current evidence supports the use of NVPs as a second-line therapy for treating tobacco use and dependence (2) after the established route of combination nicotine replacement therapy (i.e., long-acting transdermal, rapid-acting oromucosal) with behavioural counselling has been attempted (3). Despite this, public sentiment studies show regulated NVP-facilitated cessation to be an acceptable approach to tobacco cessation (4, 5) despite some health organisations holding cautious positions regarding the evidence and safety (6, 7).

Australians have access to NVPs through a variety of sources despite this being one of the most highly regulated environments for these products. The legally accepted route for accessing NVPs is through a medical prescription for purchase access or personal importation, specifically for tobacco cessation purposes. However, the more commonly used route is to identify non-legal sources for NVPs due to the relatively fewer barriers in obtaining devices this way. Importantly, the public is also exposed to a variety of media addressing known and potential health risks of NVP use (8), which can inform these types of consumer health behaviours. Recent preventive health campaigns describe the potential physical health and addiction risks associated with vaping amongst youth (9).

The physical health of people with mental health conditions is a priority in Australia’s National Preventive Health Strategy (10) given their markedly higher smoking prevalence than the wider population at 20.2% versus 9.9% of those without one in 2019 (11). Moreover, 21.0% of Australians reported a mental health condition in the past 12 months (12), including anxiety (16.8%) and affective disorders (8.0%) such as depressive episodes (4.6%) (13). Together, this indicates a population at high risk of tobacco-related illnesses.

Mental and physical illness can increase our attention to health concerns as uncontrollable contributing to reduced motivation and unhealthy behaviour (14). For example, models of depression emphasise low likelihood of positive outcomes and high probability of aversive outcomes—or pessimistic judgement bias (15). Depression and anxiety can lower quitting intentions particularly for depression (16). As depression (17) and anxiety (18) are also associated with poorer adherence to current smoking cessation approaches (e.g., transdermal patches), acceptability of alternatives, such as NVPs, warrant exploration in relation to these mental health factors.

Perceived health risks of NVP use can be influenced by socioeconomic gradient, gender, ethnicity, and sexual orientation (19), as well as by mental health status (20, 21). A study in England found that sizable proportion of people who used e-cigarettes still considered these to be equally (35%) or more (6%) (2) addictive than tobacco cigarettes with 77% reporting that they felt addicted to vaping (22). These perceptions may also influence willingness to engage with healthcare professionals who themselves weigh the prescribing risks regarding NVP safety (23). Early US research found that prescription medications can be judged as riskier than over-the-counter medications for reasons including drug familiarity, prescriber interaction, and uncertainty around health risk (24). Given these considerations, it follows that health risk perceptions of accessing NVPs via both prescription and informal routes may inform willingness to engage with this alternative to established smoking cessation methods [e.g., patch Nicotine Replacement Therapy (NRT)] among people with mental health symptoms. Understanding this further can help in framing health communication about NVP sourcing decisions and prescription access for people with anxiety and depression symptoms.

This study aims to examine how the presence of anxiety and depression symptoms may be associated with perceived health risk associated with sourcing NVPs via both routes, as well as the association of media exposure and general nicotine addiction concerns regarding NVP use. We examine the association of demographic and depression and anxiety characteristics with perceived health risk of sourcing NVPs from prescription versus non-prescription sources.

## 2 Materials and methods

### 2.1 Study design

A cross-sectional population computer-assisted telephone interview survey with the SA Health Population Health Survey Module System (PHSMS) was used. Participants were eligible if aged ≥15 years and residing in South Australia. Surveying occurred from November to December 2022. This study was part of the larger PHSMS annual omnibus telephone survey. The PHSMS used random digit dialing and multistage systematic sampling of metropolitan and regional South Australian residential centres, with approximately 3,000 respondents and up to 80 questions each year (25). Items relating to this study included question on demographics, anxiety and depression symptoms, smoking and vaping behaviour, and NVP health risk perceptions. The full bank of items can be requested from Wellbeing SA (26).

### 2.2 Participants

Of the eligible people contacted (n=3,741), non-participation (n=749) resulted from refusal (n=216), discontinued contact (n=36), physical or mental capacity (n=275), or language option unavailability (n=212). There was an 80.2% participation rate and a final sample of 3,002 respondents. Parental consent was obtained for participants aged 15 to 17 years.

### 2.3 Measures

#### 2.3.1 Sociodemographic

Sociodemographic factors included age, sex, education [range: 1 low (no schooling) to 8 high (tertiary)], and relative socioeconomic advantage in ascending deciles via socio-economic index for areas (SEIFA) (27). Primary language at home, income [range: 1 low (≤20,000) to 8 high (>150,000)], and metropolitan versus country residence were also recorded. Current smoking was determined by asking whether participants “currently smoke cigarettes, cigars, pipes, or other tobacco products” on a “daily,” “at least weekly,” “less often than weekly” basis, or “not at all.” Participants who smoked daily, at least weekly, or less often than weekly, were coded as people who smoked. Similarly, current vaping was determined by asking whether participants “currently use e-cigarettes” on a “daily,” “at least weekly,” “less often than weekly” basis, or “not at all.” Those who vaped daily, at least weekly, or less often than weekly, were coded as people who currently vaped.

#### 2.3.2 Anxiety and depression symptoms

Mental health was measured via the Patient Health Questionnaire 4 (PHQ-4), a short four-item mental health screening scale for anxiety and depression symptoms (28). Items for anxiety symptoms were as follows: 1) Over the last 2 weeks, how often have you been bothered by feeling nervous, anxious, or on edge? 2) Over the last 2 weeks, how often have you been bothered by not being able to stop or control worrying? Items for depression symptoms were as follows: 1) Over the last 2 weeks, how often have you been bothered by little interest or pleasure in doing things? 2) Over the last 2 weeks, how often have you been bothered by feeling down, depressed, or hopeless? Items are rated 0 *not at all* to 3 *nearly every day* summing two items each for depression and anxiety (range: 0–6, cutoff ≥3) symptom severity. Binary variables were created for PHQ-4 indicating likely presence of depression and anxiety. Overall mental health symptom severity score ranged from 0 to 12, with symptom cutoffs of 0–2 none, 3–5 mild, 6–8 moderate, and 9–12 severe.

#### 2.3.3 Risk perception

The perceived addiction risk of vaping was recorded by asking, “If a person uses a nicotine e-cigarette or vaping device to reduce or quit smoking, how much risk of addiction to vaping is there after they stop smoking tobacco?,” rated 1 no risk to 5 extreme risk or do not know or prefer not to say. This was categorised as 1 low, 2 moderate, or 3 high for the regressions. Perceived health risk exposure when using NVPs obtained via the non-prescription versus prescription sources was recorded by asking, “If a person is using a non-prescription nicotine e-cigarette or vaping device to reduce or quit smoking, how much health risk do you feel they are exposed to?” and “If a person is using a prescription nicotine e-cigarette or vaping device to reduce or quit smoking, how much health risk do you feel they are exposed to?” Both items were rated from 1 no risk to 5 extreme risk or do not know or prefer not to say. Exposure to vaping-related media was recorded via the item, “How often do you see media that presents scientific evidence about the use of e-cigarette or vaping devices?,” rated from 1 never (e.g., every 6 months) to 5 very frequently (e.g., multiple times per week).

### 2.4 Procedure

Project approval was obtained from the Department for Health and Wellbeing Human Research Ethics Committee (HREC/18/SAH/78/AM10), and all participants provided informed consent. An external research provider contacted participants by random digit dialing, provided a study summary, and sought consent for surveying by phone. Parental consent was obtained for those aged <18 years to participate. Languages other than English, and male or female interviewers could be chosen, where interviewers were available for this.

## 3 Data analysis

Descriptive analyses were conducted with R v.4.1.3, (29) using the packages “corrplot,” (30) “psych,” (31) and “olsrr” (32) to inspect diagnostic plots with weighted robust linear regression performed using the “robustbase” (33) package for outlier and normality considerations. Two linear regression models were fitted for the association of PHQ-4 anxiety and depression symptom presence with level of perceived health risk of prescription versus non-prescription NVPs, including variables for age, sex, social advantage, primary language, education, income, and metropolitan versus country residential classification. Interaction terms were included for tobacco smoking, vaping, and media exposure to vaping-related media, with indicators for presence of anxiety and depression symptoms, as use of nicotine products and media exposure related to this is highly likely to influence risk perceptions related to NVP use.

Key variables in the models included tobacco smoking, use of NVPs, exposure to media that presents scientific evidence about the use of e-cigarette or vaping devices, perceived risk of addiction to vaping once a person stops smoking tobacco, PHQ-4-indicated anxiety and depression symptoms, and interactions of tobacco smoking, vaping, and exposure to media about e-cigarettes and vaping, with anxiety and depression symptoms. To avoid error rate inflation, false discovery adjusted p-values using “p.adjust” in the “stats” (34) package are reported with unadjusted values and confidence intervals. Data were weighted by inverse selection probability, then by age group, by sex, and by metropolitan or country residence using 2016 Australian Census data for consistency with previous PHSMS datasets. Figures are weighted unless otherwise specified. This study was not preregistered, as no hypotheses were tested in this exploratory analysis (35).

## 4 Results

Among the participants who proceeded beyond the demographic component of the survey, those stating they were not familiar with NVPs were excluded from answering questions related to them (n=401). This exclusion led to approximately 13% of missing data for the items used in the two regression models. Selection of the items “prefer not to say” and “don’t know” accounted for further reduction in cases included in both models. A small number of influential cases were downweighted in “robustbase” based on Mahalanobis distance for the first (n=33) and second regression (n=40).

### 4.1 Sociodemographic

Sociodemographic, nicotine use, and mental health characteristics in **Table 1** show that the overall sample (N=3,002) averaged 47.90 ± 18.97 years of age, around half were female (50.82%), lived mostly in metropolitan areas (70.73%), and were well educated with a medium level of average social advantage. Low levels of current tobacco smoking (11.16%) and e-cigarette use (3.70%) were seen with many viewing vaping as a nicotine addiction risk (81.81%) and half (54.91%) reporting exposure to media that presents scientific evidence about the use of e-cigarette or vaping devices. Most participants reported no PHQ-4 mental health symptoms (69.57%), while the remaining participants reported mild (18.07%), moderate (7.10%), or severe (5.26%) overall mental health symptoms. PHQ-4 scores indicated anxiety (16.90%) and depression (12.00%) symptoms for some participants.

**Table 1:** Sociodemographic and mental health characteristics of total sample, aged ≥15 years (N=3,002).

Characteristic	Mean ± SD, *N* (%)
Age (years)	47.90 ± 18.97
Sex (male)	1,526 (50.82)
Social advantage (decile)	5.10 ± 2.94
English language (primary)	2,545 (84.78)
Education	
No schooling	3 (0.10)
Some primary school	5 (0.15)
Completed primary school	22 (0.73)
Some high school	646 (21.58)
Completed high school	408 (13.62)
Trade/certificate	456 (15.23)
Diploma/advanced diploma	332 (11.08)
University/tertiary	1,123 (37.5)
Residential area	
Metropolitan	2,123 (70.73)
Country	879 (29.27)
Currently smokes tobacco	
Yes	335 (11.16)
No	2,666 (88.80)
Currently vapes	
Yes	96 (3.70)
No	2,504 (96.29)
Considers vaping a nicotine addiction risk	
Yes	2,128 (81.81)
No	25 (0.95)
Don’t know/prefer not to say	448 (17.23)
Vaping media exposure	
Yes	1,428 (54.91)
No	1,102 (42.38)
Don’t know/prefer not to say	70 (2.71)
Anxiety symptoms^a^	
Yes	499 (16.90)
No	2,455 (83.10)
Depression symptoms^b^	
Yes	352 (12.00)
No	2,579 (88.00)

Weighted values and valid proportions shown with weighted frequencies rounded to integer and values to two decimal places. ^a^PHQ-4 anxiety ≥3, ^b^PHQ-4 depression ≥3.

### 4.2 Perceived health risk of NVPs from prescription sources for tobacco cessation

**Table 2** shows the associations between demographic, nicotine use (i.e., smoking, vaping), nicotine addiction risk perception, and anxiety and depression characteristics with perceived health risk of using prescription NVPs. The model indicated that vaping (beta=−0.732, 95% CI=−0.984, −0.480), but not smoking (beta=−0.214, 95% CI=−0.414, −0.015), was associated with lower perceived health risk of prescription NVPs. Exposure to media presenting scientific evidence about vaping was not associated with perceived prescription NVP risk (beta=−0.128, 95% CI=−0.002, 0.257). However, perceived risk of nicotine addiction from vaping to quit smoking was associated with higher perceived health risk of prescription NVPs (beta=0.784, 95% CI=0.690, 0.787).

**Table 2:** Robust linear regression summary for demographic factors and presence of anxiety and depression symptoms predicting perceived health risk associated with accessing NVPs through prescription sources (N=1,336).

Effect	*Beta*	SE	95% CI LL	95% CI UL	*t*	*p*	*p_adj_*
Intercept	1.342	0.255	0.842	1.842	5.269	**<0.000**	**<0.000**
Age (years)	0.003	0.002	−0.001	0.007	1.646	0.100	0.200
Gender (male)	−0.082	0.062	−0.204	0.041	−1.305	0.192	0.329
Social advantage (decile)	−0.030	0.012	−0.054	−0.007	−2.499	**0.013**	**0.050**
English language (primary)	0.196	0.099	0.002	0.390	1.977	**0.048**	0.135
Education (rank)	−0.009	0.020	−0.047	0.030	−0.432	0.666	0.701
Income (rank)	−0.004	0.014	−0.033	0.024	−0.310	0.757	0.757
Metropolitan (yes)	0.042	0.063	−0.082	0.167	0.663	0.507	0.597
Smokes (yes)	−0.214	0.102	−0.414	−0.015	−2.111	**0.035**	0.116
Vapes (yes)	−0.732	0.128	−0.984	−0.480	−5.701	**0.000**	**<0.000**
Media exposure (yes)	0.128	0.066	−0.002	0.257	1.929	0.054	0.135
Addiction risk (low/mod/high)	0.784	0.048	0.690	0.877	16.434	**<0.000**	**<0.000**
Anxiety symptoms	0.067	0.127	−0.183	0.318	0.529	0.597	0.663
Depression symptoms	0.126	0.123	−0.116	0.368	1.021	0.308	0.439
Smokes (yes)* Anxiety symptoms (yes)	−0.444	0.240	−0.914	0.026	−1.853	0.064	0.142
Vapes (yes)* Anxiety symptoms (yes)	−0.309	0.240	−0.779	0.161	−1.290	0.197	0.329
Media exposure (yes) * Anxiety symptoms (yes)	0.129	0.160	−0.185	0.443	0.803	0.422	0.528
Smokes (yes)* Depression symptoms (yes)	0.256	0.221	−0.177	0.689	1.159	0.246	0.379
Vapes (yes)* Depression symptoms (yes)	0.700	0.249	0.212	1.188	2.813	**0.005**	**0.025**
Media exposure (yes) * Depression symptoms (yes)	−0.134	0.161	−0.449	0.181	−0.836	0.403	0.528

*R^2^* = 0.341, Adjusted *R^2^* = 0.332, model fit: *χ^2^_diff_* (19, N=1,335)=56.151, p<0.001.

CI, confidence interval; LL, lower limit; UL, upper limit. Social advantage expressed as SEIFA decile score, with higher score indicating greater social advantage. For education (scored 1 *low* to 8 *high*) and income (scored 1 *low* to 9 *high*), see Measures. Practically significant *p*-values are bolded. *p_adj_* = Benjamini and Hochberg correction.

Bolded values indicate p-values of interest.

Mental health symptoms were not associated with health risk views on prescription NVPs when indicating presence of anxiety (beta=0.067, 95% CI=−0.183, 0.318) or depression (beta=0.126, 95% CI=−0.116, 0.368). Associations of smoking, vaping, and media exposure with health risk views on prescription NVPs were not modified by the presence of anxiety symptoms. Similarly, associations of smoking and media exposure with health risk views on prescription NVPs were not modified by the presence of depression symptoms. However, vaping was associated with a higher perceived risk of prescription NVPs among people with depression symptoms (beta=0.700, 95% CI=0.212, 1.118) (see **Table 2**).

### 4.3 Perceived health risk of NVPs from non-prescription sources for tobacco cessation

**Table 3** shows the associations between demographic, nicotine use (i.e., smoking, vaping), nicotine addiction risk perception, and anxiety and depression characteristics with perceived health risk of using non-prescription NVPs. Both vaping and smoking were associated with lower perceived health risk of non-prescription NVPs with this being stronger for vaping (beta=−0.661, 95% CI=−1.196, −0.126) than for smoking (beta=−0.310, 95% CI=−0.558, −0.062). Exposure to media on scientific evidence about vaping was not associated with perceived non-prescription NVP risk. Perceived risk of nicotine addiction from vaping to quit smoking was associated with higher perceived health risk of non-prescription NVPs (beta=0.733, 95% CI=0.602, 0.865).

**Table 3:** Robust linear regression summary for demographic factors and presence of anxiety and depression symptoms predicting perceived health risk associated with accessing NVPs through non-prescription sources (N=1345).

Effect	*Beta*	SE	95% CI LL	95% CI UL	*t*	*p*	*p_adj_*
Intercept	1.646	0.309	1.039	2.253	5.321	**<0.000**	**<0.000**
Age (years)	0.004	0.003	−0.001	0.009	1.421	0.156	0.370
Gender (male)	−0.057	0.064	−0.182	0.068	−0.898	0.369	0.518
Social advantage (decile)	−0.034	0.012	−0.056	−0.011	−2.939	**0.003**	**0.022**
English language (primary)	0.022	0.081	−0.138	0.182	0.271	0.786	0.844
Education (rank)	−0.004	0.019	−0.041	0.034	−0.197	0.844	0.844
Income (rank)	0.020	0.015	−0.008	0.049	1.384	0.167	0.370
Metropolitan (yes)	0.061	0.077	−0.091	0.212	0.789	0.430	0.538
Smokes (yes)	−0.310	0.126	−0.558	−0.062	−2.456	**0.014**	**0.062**
Vapes (yes)	−0.661	0.273	−1.196	−0.126	−2.423	**0.016**	**0.062**
Media exposure (yes)	0.037	0.072	−0.104	0.177	0.511	0.609	0.717
Addiction risk (low/mod/high)	0.733	0.067	0.602	0.865	10.963	**0.000**	**<0.000**
Anxiety symptoms	0.115	0.107	−0.094	0.325	1.080	0.280	0.518
Depression symptoms	−0.107	0.105	−0.313	0.098	−1.025	0.306	0.518
Smokes (yes)* Anxiety symptoms (yes)	−0.291	0.337	−0.951	0.370	−0.863	0.388	0.518
Vapes (yes)* Anxiety symptoms (yes)	−0.247	0.281	−0.798	0.303	−0.881	0.378	0.518
Media exposure (yes) * Anxiety symptoms (yes)	−0.029	0.148	−0.319	0.261	−0.197	0.843	0.844
Smokes (yes)* Depression symptoms (yes)	0.323	0.218	−0.104	0.750	1.483	0.138	0.370
Vapes (yes)* Depression symptoms (yes)	0.550	0.331	−0.099	1.200	1.664	0.096	0.321
Media exposure (yes) * Depression symptoms (yes)	0.141	0.150	−0.153	0.435	0.940	0.348	0.518

*R^2^* = 0.353, Adjusted *R^2^* = 0.344, model fit: *χ^2^_diff_* (19, N=1,344)=28.132, p=0.081.

CI, confidence interval; LL, lower limit; UL, upper limit. Social advantage expressed as SEIFA decile score with higher score indicating greater social advantage. For education (scored 1 *low* to 8 *high*) and income (scored 1 *low* to 9 *high*), see Measures. Practically significant *p*-values are bolded. *p_adj_* = Benjamini and Hochberg correction.

Bolded values indicate p-values of interest.

Mental health symptoms were again not associated with risk views on non-prescription NVPs when indicating presence of anxiety (beta=0.115, 95% CI=−0.094, 0.325) or depression (beta=-0.107, 95% CI=−0.313, 0.098). However, associations of smoking, vaping, and media exposure with risk views on non-prescription NVPs were not modified by the presence of anxiety or depression symptoms.

## 5 Discussion

This study found that there is little difference between how people view health risk of vaping-facilitated tobacco smoking cessation when NVPs are sourced via medical prescription versus non-prescription sources (e.g., other people, illegal sales). This latter category encompasses illegal NVP markets, a source of vaping products that Australia is moving to further restrict access to (36). As this leaves only prescription access for smokers opting for NVP-facilitated cessation, preventive health communications about this process consider how mental health symptoms can factor into this process.

Concern about the potential risk of nicotine addiction from vaping being related to increased health risk concern about using NVPs from either source is consistent with other findings that stronger perceptions of addictiveness are inversely related to the use of e-cigarettes (37).

Our study identified no main effect of anxiety or depression on perceptions of health risks of NVP-facilitated smoking cessation—whether via the prescription or non-prescription route. However, a key finding related to depression symptoms and being a person who vapes. Findings showed that being either a person who smokes or who vapes was associated with seeing lower health risk in NVP-facilitated quitting, irrespective of sourcing NVPs via prescription or elsewhere. Though when mental health symptoms indicated depression in a person, being a person who uses a vape was associated with seeing greater health risk in NVP-facilitated quitting—though only for prescription-sourced NVPs.

If this effect is meaningful—and further research is necessary to test this—it may mean that people experiencing depression who vape have a low likelihood of engaging with the prescription access system for quitting—something they may benefit from in the future if availability of non-prescription NVPs becomes more difficult under tightened Australian tobacco and vaping regulations. Alternatively, this finding could arise from the assumption that the way a person currently accesses NVPs will continue to be available in the future. As noted, there is a low likelihood that this source is prescription access given research showing proportions for this (7%) compared to other sources (e.g., vape shop, 53%; friend over 18, 39%; tobacconist, 33%; online, 30%) (37).

A further possible reason could relate to pessimistic judgement bias present in models of depression (15), where a person might anticipate poorer outcomes regarding health risks of prescription access NVPs. This may limit intentions to use this approach for smoking cessation or for switching from non-prescription to prescription NVPs. Studies of clients of psychiatric services indicate complex relationships with nicotine use and cessation, including overall declining health and pessimism about success of nicotine replacement (38). Australia has mental health-specific support for quitting smoking (39, 40), a suitable avenue for addressing barriers to NVP-facilitated cessation by people living with depression.

It may also be the case that population-specific supported or subsidised access for people experiencing depression, coupled with targeted education on health risk evidence, may support access to a wider range of cessation pharmacotherapies in this group. As with tobacco cessation (41), health messaging focused on benefits to mental health could be tested, though in this case in relation to the benefits of accessing or switching to prescription NVPs versus via other unregulated sources.

## 6 Limitations

Limitations to the current study included, first, that a large number of respondents selected opt-out responses for key variables (i.e., “I don’t know,” “prefer not to say”). This was seen, for example, with considering vaping a nicotine addiction risk. However, excluding this, respondents still provided sufficient data for the models to be run. Second, as the overall sample was structured to be representative of the South Australian population, the prevalence of vaping, tobacco smoking, and respondents with high anxiety depression symptoms was relatively low. Third, the item used to ask about “exposure to scientific evidence about the use of e-cigarettes’” did not account for individual level of scientific literacy as a factor in the perceived and actual robustness of evidence that respondents considered for this item. Extensions of this study should factor in a measure of scientific literacy. Further research is needed that solely recruits people with clinically meaningful anxiety or depression symptoms, and potentially symptoms of other conditions. Fourth, both models should be replicated with a national Australian sample and be paired with qualitative comments to capture why people may see differing levels of addiction risk for both NVP source categories.

## 7 Conclusions

This study provides a brief and exploratory snapshot of how mental health symptoms of South Australians may affect perceptions of health risk of the NVP prescription access model versus via other non-prescription sources. General concern about nicotine addiction risk in NVP-facilitated smoking cessation was related to heath concerns of NVP use, irrespective of prescription or non-prescription source. People who vape and experience depression symptoms may see greater health risk in using the NVP prescription access model compared with those without depression symptoms. This has potential implications for this priority population in accessing prescription-sourced NVPs as part of a wider range of smoking cessation pharmacotherapies given that non-prescription sources are more highly restricted in Australia. Qualitative interviews with tobacco smokers with and without clinically meaningful anxiety or depression symptoms are needed to identify how health risk perceptions in this population can inform mental health-tailored smoking and vaping cessation programs.

## Data availability statement

The data analysed in this study is subject to the following licenses/restrictions: Permission is required from Wellbeing SA and SA Health to access this South Australian population health data (42). Requests to access these datasets should be directed to joshua.trigg@flinders.edu.au.

## Ethics statement

This study was approved by SA Health Department for Health and Wellbeing Human Research Ethics Committee (HREC/18/SAH/78/AM10). The study was conducted in accordance with the local legislation and institutional requirements. Informed consent for participation in this study was provided by participants, and was provided by participants’ legal guardians were required.

## Author contributions

JT: Conceptualization, Data curation, Formal Analysis, Methodology, Writing – original draft, Writing – review & editing. RC: Conceptualization, Investigation, Writing – original draft, Writing – review & editing. PA: Conceptualization, Writing – review & editing. JB: Writing – review & editing. BB: Conceptualization, Writing – original draft, Writing – review & editing.
